# How do Swiss medical schools prepare their students to become good communicators in their future professional careers: a questionnaire and interview study involving medical graduates, teachers and curriculum coordinators

**DOI:** 10.1186/s12909-018-1376-y

**Published:** 2018-11-29

**Authors:** N. Junod Perron, C. Klöckner Cronauer, S. C. Hautz, K. P. Schnabel, J. Breckwoldt, M. Monti, S. Huwendiek, S. Feller

**Affiliations:** 1 Unit of Development and Research in Medical Education, Faculty of Medicine, University of Geneva, Rue Michel Servet 1, 1211 Genève 4, Switzerland; 2 Department of Emergency Medicine, Inselspital University Hospital, University of Bern, Bern, Switzerland; 3 Institute of Medical Education, University of Bern, Bern, Switzerland; 4 Office of Dean, Faculty of Medicine, University of Zurich, Zurich, Switzerland; 5 Unité pédagogique, Faculty of Biology and Medicine, University of Lausanne, Lausanne, Switzerland

**Keywords:** Swiss, Communication, Curriculum, Medical students, Training

## Abstract

**Background:**

Since 2011, the Swiss Catalogue of Learning Objectives (SCLO) has provided the framework for assessing communication skills in the Swiss Medical Federal Licensing Examination (FLE). This study evaluates how far the communication curricula of five Swiss medical schools match the SCLO and international recommendations. It also explores their strengths, weaknesses, opportunities and threats (SWOT).

**Methods:**

A mixed method approach was used. In a first step, curriculum coordinators/key communication skills teachers and medical graduates were asked to fill out a questionnaire based on communication related objectives from the SCLO and a review of European consensus statements on communication training. Second, information was collected from all Swiss medical schools to identify which communication skills were taught in which formats and at what time points within the 6-year curricula. Finally, 3–4 curriculum coordinators/key communication skills teachers from each medical school were interviewed about their communication curriculum, using SWOT analysis.

**Results:**

Sixteen teachers/coordinators (response rate 100%) and 389 medical graduates (response rate 43%) filled out the questionnaire. Both the teachers/coordinators and the graduates considered that two thirds of the communication items listed in the questionnaire were covered in their curricula. Between sixty and two hundred structured hours were dedicated to communication, predominantly in small group and experiential formats. Assessment relied on both MCQs and OSCEs. Most of the training occurred during the first three years of medical school. Teachers felt that the need for communication skills training was now well-recognized by their institution and was taught with appropriate teaching methods. However, recruitment and training of teachers, continuity of communication skills training during clinical years, and the adoption of a common frame of reference among the five medical schools, remained a challenge.

**Conclusion:**

Although the Swiss medical schools all offered a partly longitudinal communication skills training, with appropriate teaching methods, this study indicates that the communication skills actually taught do not fully match the SCLO or international recommendations. There was less training for complex communication skills training during the clinical years, and ensuring quality and coherence in the teaching remained a challenge.

**Electronic supplementary material:**

The online version of this article (10.1186/s12909-018-1376-y) contains supplementary material, which is available to authorized users.

## Background

The use of appropriate communication skills has a significant impact on patient care, and is correlated with several improved patient and healthcare outcomes ranging from patient satisfaction, patients’ self-management, the consultation process, physician time management and health behaviours, to the human, medical-legal and economic costs of care [1–5].

Teaching and assessing communication skills are recognized as essential subjects in medical schools, and national and supranational medical organisations in various countries have published guidelines to support the development of communication skills in the curricula [6–12]. Similarly, several recommendations have been made regarding communication skills training, based on evidence and observations. Teaching of these skills should be longitudinal, in order to improve skill retention, as students’ communication skills tend to decline as they progress through clinical training [13–15]; it should include learner-centered and experiential instructional methods such as role-play, practice with simulated patients, observation followed by feedback, and small group discussions, all of which are effective [16, 17]. Communication skills training should be integrated into the overall medical curriculum and the practical clinical training activities, including those for the different medical specialties [18–20]. Finally, more attention should be paid to the quality of workplace-based communication skills training during clinical years, as this training usually occurs informally, opportunistically, and essentially through implicit role modelling. Observation followed by feedback is rarely reported, and when it does take place, the feedback is often described as vague, unspecific and focused on content more than on communication [21–23].

Communication skills training programs have been integrated into the undergraduate medical curricula of many countries in recent decades. Several national or supranational studies investigating the implementation of such programs showed wide variations among programs, regarding the skills covered, time spent on the topic, types of training session, and formats of assessment [24, 25]. Although some flexibility in communication skills training is acceptable, all the medical schools in a particular country should offer comparable training in the core dimensions of communication, especially when communication is assessed in a national licensing examination [24].

In Switzerland, the Swiss Catalogue of Learning Objectives (SCLO), published in 2002 and revised in 2008, sets up a definition of the objectives for undergraduate medical education [26]. Among others, it lists twenty-one learning objectives under the section “Communication”. It also specifies the objectives of the Federal Licensing Exam (FLE) that follows undergraduate medical training, and defines the competences medical candidates should have acquired before entering postgraduate training. Since 2011, the Swiss FLE has assessed communication skills together with medical knowledge and skills in a twelve-station OSCE conducted at the end of medical school [27].

Apart from the existence of the SCLO and the FLE, each medical school has full autonomy in organizing its medical curriculum and adapting it over time, according to new developments in medical education and institutional priorities. Thus, the extent to which undergraduate medical training integrates communication and is in line with international recommendations may vary from one medical school to another.

In 2014, the Swiss Federal Office of Public Health commissioned a project aimed at improving the assessment of communication skills in the FLE in Human Medicine. The first step of a 5-step project was to evaluate how medical schools train and assess communication skills in their own curricula. We were especially interested in (1) evaluating how far the communication curricula of the five medical schools matched the SCLO, (2) determining whether the communication curricula structure, the instruction and the assessment methods used were in line with international recommendations, and (3) exploring coordinators’ and teachers’ views on the development and implementation of their own communication curricula, using a “SWOT” analysis.

## Methods

### Design and setting

**Fig. 1:**
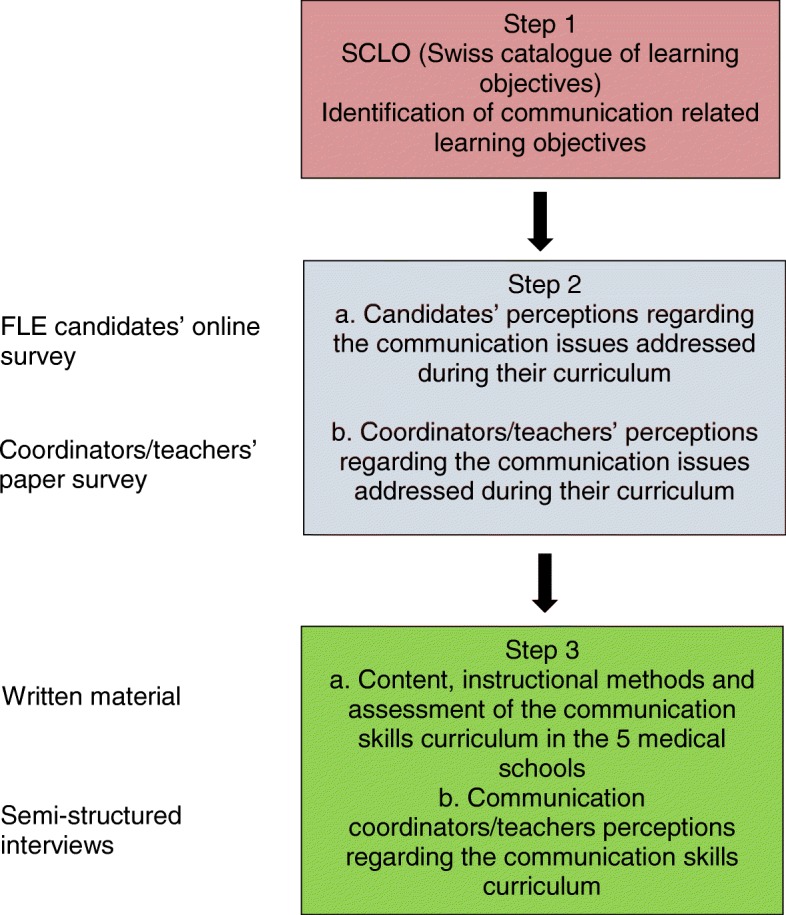
Study design

We conducted a mixed-method study between September and December 2015 at all five medical schools in Switzerland offering a complete curriculum (Basel, Bern, Geneva, Lausanne and Zürich). The study included surveys, analysis of written material, and semi-structured interviews (3) (Fig. 1).

### Participants and procedure

#### Step 1: - Identification of communication related learning objectives

To evaluate the match between FLE, SCLO and the content of the medical schools’ communication curricula, we initially identified all the communication-related learning objectives listed in the different sections of the SCLO. Bearing in mind that the communication curricula could also address communication issues not mentioned in the SCLO, we compared them to the learning objectives of the European and Basel consensus statements about communication training [6, 7]. We obtained a list of 33 learning objectives related to the main communication issues that could be taught during undergraduate medical training and assessed through OSCEs.

#### Step 2: - Candidates’ and coordinators/teachers’ perceptions regarding the communication issues addressed during their curriculum

We then developed a 33-item questionnaire asking the candidates to state which of the items they had received training in, at their own medical school and to which extent they felt prepared for communication in future professional life (1 additional item) (step 2a) (Table 1). The content validity evidence was tested using the established literature. The response process validity evidence was established using a think aloud process involving four French-speaking and three German-speaking medical students, which led to improvements of question formats and content. The questionnaire was sent out as part of a regular online survey for feedback of the FLE 2015, which was sent to all medical candidates (*n* = 915) on the day of the FLE. It was filled in anonymously and voluntarily by the candidates. They were asked to answer the question “what communication skills are taught?”, marking “Yes”, “No” or “I don’t know” for the 33 items listed and scoring 1–5 for the overall degree of preparedness regarding communication skills (1 item). An electronic follow-up reminder was sent once, four weeks after the questionnaire was initially sent out. The same questionnaire was filled in by two or three key communication training teachers, and one curriculum coordinator of each medical school during a semi-structured interview (step 2b). The teaching professionals answered the same questions apart from the one about preparedness, but scored the 33 items on a 1–5 Likert scale (Table 1).

**Table 1:** Teachers’ and candidates’ perceptions regarding communication skills addressed during the undergraduate curriculum (candidates’ n ranged from 305 to 389; teachers/coordinators *n* = 16)

Topics of the Swiss catalogue of learning objectives	Teachers’ perceptions	Candidates’ perceptions	
	Likert 1–5	% Yes	Responding
	Mean (SD)		
Generally covered topics			
Patient-focused history taking			
− Focused	4.7 (0.5)	94.1	389
− Reasons	4.8 (0.4)	97.9	384
− Expectations	4.2 (0.9)	87.4	357
− Somatic-psychological aspects	4.6 (0.6)	95.9	365
− Concerns	4.2 (1.0)	88.6	367
− Socio-cultural background	4.2 (0.7)	84.0	356
− Illness experience	3.8 (0.9)	78.2	339
Ask patient needs regarding health problems	4.1 (0.9)	73.2	321
Disease-illness concepts	4.0 (1.3)	86.3	342
React to nonverbal cues	4.1 (0.9)	76.2	319
Balance proximity-distance	3.9 (1.1)	62.6	305
Show empathy	4.2 (1.0)	86.8	319
Establish and maintain an empathetic relationship	4.5 (0.5)	74.8	314
Give time to patient	4.8 (0.4)	98.1	371
Active listening	4.8 (0.5)	96.2	366
Collecting information about delicate issues (sexual)	4.1 (1.0)	79.1	358
Obtaining patient consent	3.8 (1.1)	82.5	337
Explaining reasons for investigation, risks, advantages	3.6 (1.0)	82.9	333
Explaining and checking patient understanding	4.3 (0.8)	84.9	325
Breaking bad news	4.1 (0.9)	80.9	330
Counselling skills	3.9 (1.0)	88.4	327
Generally less covered topics			
History taking in absence of patient	2.4 (1.4)	62.6	372
History taking with special needs patients (dying)	3.3 (1.1)	59.5	346
Communicating with patients with language problems	1.8 (1.0)	21.2	344
Communicating with allophone patients	3.0 (1.3)	45.7	352
Communicating with vulnerable patients	2.9 (1.2)	63.9	341
Phone conversation	2.5 (1.6)	53.5	368
Document information	2.5 (1.2)	39.9	326
Shared decision making	3.3 (1.3)	66.6	323
Use of visual support	2.2 (0.9)	49.2	309
Managing unsatisfied or unhappy patients/families	2.4 (1.3)	43.3	321
Interprofessional communication	2.4 (0.9)	48.7	318
Presentation skills	3.4 (1.1)	62.2	315

#### Step 3a: - Communication curricula of the Swiss medical schools

In relation to the content of the five communication curricula, one investigator per medical school asked via email or phone the coordinators of the communication curricula in each of the five medical schools to send us comprehensive information regarding their own, specific communication curriculum. It was checked with the available information of the website of each medical school. Information was then categorized according to the following elements: academic year, learning objectives, topics, instructional methods, teachers’ profile, and assessment. Data were summarized in the tables. Any necessary clarification or additional elements were obtained orally, before submitting the final tables to the coordinators for validation.

#### Step 3b:-Communication coordinators/teachers perceptions regarding the communication skills curriculum

Finally, coordinators’ and teachers’ perceptions and perspectives regarding the communication skills taught in their own settings were explored through semi-structured interviews. The interview guide included questions about teachers’ and coordinators’ sociodemographic characteristics, the curricular history in the teaching of communication, and finally, the strengths (S), weaknesses (W), opportunities (O) and threats (T) of their communication curricula (i.e. a SWOT analysis). The interviews were audio recorded and transcribed verbatim.

The study was granted a waiver from the need for approval by the Ethical Committee of the Canton of Geneva, as it did not involve the collection of any personal health information (article 2 of the Swiss Federal Act on Research involving Human Beings) [28]. The participants were informed that the data would be analysed and reported (after being anonymized, in the case of the teachers and coordinators).

### Analysis

Data from the surveys were analyzed descriptively using means and percentages and statistically using SPSS 24. To evaluate the match between candidates’ and teachers’ coordinators’ answers, we calculated the difference in the rank order of candidates’ yes answers and teachers/coordinators Likert scale answers using the paired Wilcoxon rank sum test. A thematic analysis of the semi-structured interviews was performed. After reading and discussing the content of 10 transcripts, two individual researchers (NJP and CKC) defined codes for the following themes: institutional recognition, frame of reference, curricular organisation (including structure, instructional methods and teachers), evaluation/assessment, and resources. The researchers then conducted a SWOT analysis on the curricula. CKC coded all the transcripts. The coding was cross-checked for French transcripts by NJP and for German transcripts by SCH. Any divergences between them were discussed until consensus was reached.

## Results

From a total of 915 FLE candidates, 606 gave feedback to the FLE (exam specific, not part of this study) and of those who gave feedback, 389 filled out the questionnaire on communication (response rate: 43%). Eleven teachers and 5 curriculum coordinators answered the survey and were interviewed: 12 male, 4 female; between 44 to 63 years old (M = 52.8, SD = 5.78); the mean number of years of clinical practice was 20.9 (SD 10.65) and the mean number of years of CS teaching was 9.1 (SD 7.30).

### Match between communication-related SCLO/European statements and content of the communication curricula

Table 1 shows that about two thirds of the learning objectives were covered during undergraduate medical training (> 3.5 on Likert scale or > 60%). Candidates felt globally moderately prepared for communication in future professional life (Likert scale 1–5, 1 = low; 5 = high mean 3.8 (SD 0.83)). There were disparities between medical schools in terms of the number of topics covered: one medical school did not prepare their candidates for most of the items listed, while two medical schools only partially covered the topics (Additional file 1). Such disparities were reflected in the candidates’ perceived preparedness for communication in professional life. The communication curricula focused essentially on the basics of clinical communication, such as history taking, patients’ perspectives, empathy, listening, explaining, counseling and checking understanding. Issues less covered during the communication training related to communication with special patient groups (vulnerable patients, allophones, children), shared decision making, dealing with dissatisfied patients, interprofessional communication, and oral presentation skills. The teachers’ perceptions about communication skills training matched the candidates’ perceptions (Wilcoxon Z = − 0.17, *p* = 0.865).

### Organization of the different communication curricula

All the medical schools reported having a specific communication curriculum based on different frameworks for its development and implementation. One relied on the Basel consensus, the Calgary Cambridge and the CanMEDS frameworks, while two used the Calgary Cambridge guide only. The two remaining medical schools reporting using the SCLO as a basis.

**Fig. 2:**
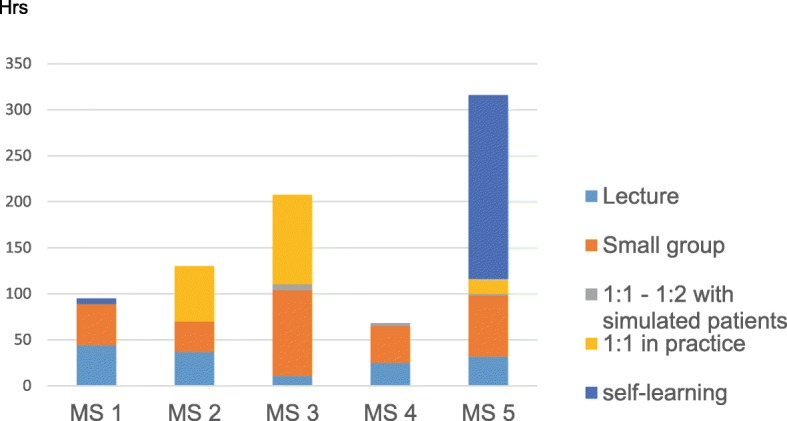
Number of hours dedicated to communication training in the five Swiss medical schools (MS = Medical School)

**Fig. 3:**
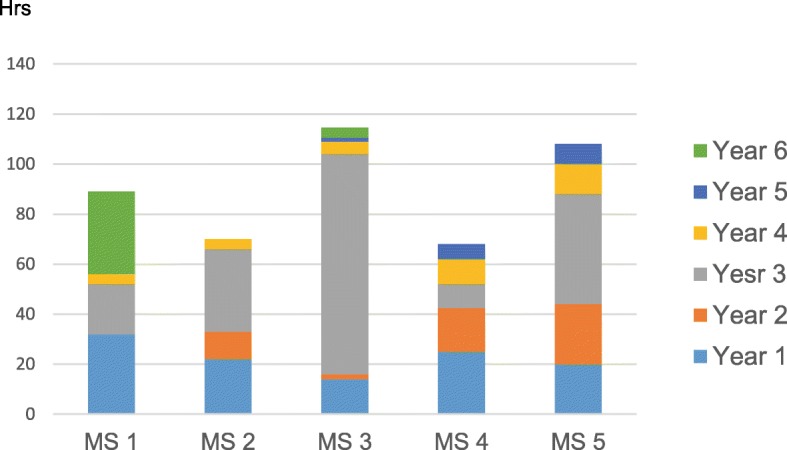
Distribution of structured hours dedicated to communication training in the five Swiss medical schools (MS = Medical School)

The number of reported structured hours dedicated to communication training was heterogeneous, ranging from 60 to 200 h. Instructional methods included lectures, small group work with the use of videos, role playing, interaction with simulated patients, interactions with patients in general practices under the supervision of general practitioners, and interactions with simulated patients in 1:1 or 2:1 training (Fig. 2). One medical school asked its students to produce a written and reflective assignment on a topic relating to the human dimensions of medicine, attributing 200 h of self-directed learning to this task. The training predominantly took place in 1:1 teaching in general practice settings or small groups (Fig. 2). The proportion of lectures varied, but there tended to be a higher number of lectures during the first year for most medical schools. The main structured training activities essentially took place in the 2nd and 3rd years, apart from one medical school, where several lectures were given during the 6th year (Fig. 3).

The teachers involved in the lectures on communication skills were essentially psychiatrists and to a lesser extent, those of other medical specialties, for instance internists or general practitioners. For small group or 1:1 training, the teacher profiles were more diverse, and included psychiatrists, general practitioners/internists, educationalists and to a lesser extent, professionals from other specialties.

Both MCQs and OSCEs were used for the summative assessment, but within different years of studies and varying frequencies among medical schools (no summative OSCE in one medical school). The number of stations per OSCE also varied. All the medical schools, except for one, integrated the rating scale used for the FLE during its faculty OSCEs (Table 2).

**Table 2:** Type and distribution of summative assessment of communication in the five Swiss medical schools

	MS 1	MS 2	MS 3	MS 4	MS 5
Year 1	MCQ	MCQ	–	MCQ	MCQ Oral presentation/written exam
Year 2	–	MCQ	Oral and practical exam MCQ		OSCE 5 stations MCQ
Year 3	MCQ	OSCE 4–6 stations MCQ	OSCE 16 stations	OSCE 3 stations	OSCE 5 stations
Year 4	–	OSCE 6 stations MCQ	–	OSCE 6 stations	–
Year 5	–	OSCE 5 stations	OSCE 9 stations	–	OSCE 4 stations
Year 6	–		–	–	–
Rating scale used		FLE rating scale	FLE rating scale	Self-made	FLE rating scale

*MCQ* multiple choice questions, *OSCE* objective structured clinical examination, *FLE* federal licensing exam

Workplace-based formative assessment was reported in all five medical schools during the clinical years. However, they did not specifically focus on communication issues; they took different forms (self-made, mini CEX,…); and the required number of assessments differed. The supervisors were mainly clinicians not specifically trained in communication or involved in communication skills teaching.

### Communication teachers’ and coordinators’ perceptions regarding their communication curriculum

The main outcomes of the content analysis of the interviews are presented below, with some exemplary statements in Table 3.

**Table 3:** Quotes extracted from the semi-structured interviews conducted with key communication skills teachers and curriculum coordinators (MS = Medical school)

Strengths	Institutional support	*Quote 1: Yes, exactly. But also, I have the feeling, at least since I started taking over, that deanery and faculty are very open to improvements, and this proposal of mine, which costs money, to use patient actors systematically, was accepted immediately. That was even heartily supported by (X and Y?) and by the Vice Dean for Education. MS1*
	Curriculum structure	*Quote 2: Somehow, we also have a structure, I think, a pedagogical structure which makes sense. We … first students are asked to observe and put words on what they observe. Then they do some role playing themselves. And then they meet patients, real patients, standardized (simulated) patients, always in run-of-the-mill situations, not necessarily too complex ones. And then, when preparing their Master’s degree, they have … they tackle more specific, more complex questions such as the announcement of a severe diagnosis or the motivation interview … this kind of things and so somewhere it … luckily, but like this it is gradual. Therefore, I believe it is rather well thought out (done).MS5*
	Framework	*Quotes 3:* *So, as I was telling you, we really have something that has been identified for this now. I think it is supported by a sufficiently widely recognized model to believe it is valid (Calgary-Cambridge Guide) and I know it is used in X, in Y. I am also in a position of responsibility here, therefore I know that it is also the model. MS4* *Well, in short, all this to say that it is not too esoteric; it is something that is known. And then, finally, when other models are mentioned to us we can very often draw a parallel with this one. MS5*
Weaknesses	Lack of continuity	*Quotes 4:* *Or to bridge this gap, somewhere we have the feeling, there is a gap somewhere during clinical years. Before they were trained and then they are completely focused on managing their mostly technical daily duties, to get it all correct and right, and so, and/ (…) MS3* *The other weakness is that there are plenty of subjects which are not taught, or else taught too early, because they are rarely taken up again later on. MS4*
	Insufficient coherence	*Quote 5: I think, it is very heterogeneous, depending on who the teacher is. We have very good documents and a solid database, but it’s just/ it’s impossible to standardize, because it varies a lot depending on which teacher is in charge, I think. MS2*
	Insufficient feedback by experienced supervisors	*Quote 6: On the one hand, of course, I see the present lack of sufficient infrastructure as a weakness, but also and above all the lack of financial and especially personnel resources. We often had to restrict ourselves to communication feedback from the perspective of the actor (standardized patient?), and would have frequently rather liked to hear experts who, being on their home ground, already, yes, bring some experience. The patient’s perspective is one thing, but I consider professional supervision, also in such communication scenarios which also have technical aspects, I think it is very/or not only me, but we think it is very, very important and we would like to intensify that. MS3*
Opportunities	Entrustable Professional Activities	*Quote 7: I would say that the chance … another opportunity is that we are currently switching from the learning objectives to the entrustable professional activities, do you know what it is? And this, I think, is clearly an opportunity, because I believe that in EPA it is actually integrated. Therefore each time, if you say ‘he must know how to explain the preoperative assessment’ there is some communication. So I think that it is in fact a great opportunity, if one introduces the EPA, to introduce communication and professionalism, and ethics and values in the whole curriculum. So this, I think is a great opportunity.MS4*
Threats	New potentially basic science oriented medical schools	*Quote 8: And of course then I am wondering, if there is now a Bachelor in medicine offered by the ETH, which doesn’t have an actual medical faculty and no patient contact, they do not have a hospital really and the ETH is an outstanding University for basic sciences and technology and they offer a tremendous amount of qualities, but physician-patient-communication (laughing) probably isn’t part of these qualities. MS3*
	Academic promotion	*Quote 9: Another (…) weakness I see, is that/But that’s, I think, a problem with teaching in general, that the real top-top-level of the faculty/I can’t say they’re not interested, that would be wrong. There are some, who are really involved, but have other priorities. So, priorities are technical development and research, but I suppose, that’s not only the case here in (...). MS3*
	Development of a reductionist vision of communication	*Quote 10: As far as weaknesses are concerned, I think that one of them is that we are still staying a little too much on the level of the recipe. It means getting technique and communication mixed up. I think that to focus our training in communication on standardized patients and the OSCE makes us feel that we are in something which is perceived as artificial… we have to start weakening (opening up) the checklist syndrome and develop more the job perspective in the wider meaning of the word. The job of a physician is the relationship, which then takes up a variety of forms. (comes in handy in lots of situations). MS5*

#### Strengths

The teachers and coordinators of all the medical schools acknowledged having received strong support from their deans of education over the years, to develop and maintain a communication curriculum. They also felt that communication training was recognized as important (Quote 1).

Some of the medical schools reported having received more support when the new FLE was introduced, with a shift from a psychosocial to a more skills-based approach to communication. Others already had a strong tradition in communication skills training, and had only slightly or moderately adapted their communication curricula and assessment methods based on the new FLE requirements.

Other strengths mentioned by the majority of teachers and coordinators related to the curricular structure. It was described as longitudinal, progressive, and moving from basic to more complex issues over the years. In most of the medical schools, communication was becoming increasingly integrated with training in other clinical skills (Quote 2).

Most of the participants also felt that the instructional methods, essentially the experiential and interactive ones, were appropriate, and that teachers from different backgrounds offered different and complementary perspectives for students.

Those medical schools that used a well-recognized framework, such as the Calgary-Cambridge framework, considered that these anchors gave coherence to their curriculum (Quotes 3). Others reported having used the SCLO communication-related learning objectives to thoroughly match and articulate all their training activities.

#### Weaknesses

Several weaknesses were reported in relation to curriculum organization. The lack of continuum in communication teaching between the pre-clinical and clinical years, or between undergraduate and postgraduate training, and the fact that there was a lack of communication training during the clinical years, were cited by the clear majority of teachers and coordinators (Quote 4).

Some of the teachers considered that there was still too little collaboration and coherence between modules, as their organization was the responsibility of different departments. Others were concerned about the heterogeneity of teachers’ experiences and expertise, and felt that additional faculty development should improve the quality of teaching (Quote 5).

In some medical schools, opportunities for students to receive feedback, or to practice under the supervision of experienced and well-trained clinicians, were still insufficient (Quote 6).

#### Opportunities

Some participants considered that the existence of an FLE was an opportunity to influence the range and variety of communication topics addressed during training, and to improve communication curricula. For others, replacing the learning objectives of the SCLO with “Entrustable Professional Activities” (EPAs) [29] in the planned new Learning Catalogue [30] was seen as a way to facilitate the integration of communication skills with other clinical skills training, and to facilitate the continuity of communication training and assessment between the pre-clinical and clinical years, and between undergraduate and postgraduate training (Quote 7).

Finally, some of the participants hoped that taking part in a federal project aiming at improving the assessment of communication skills in the Swiss FLE would be an opportunity to develop a Swiss consensus on communication skills training, involving representatives of all five medical schools.

#### Threats

The interviewed teachers and coordinators saw no current major threats, but feared that time and money may become a major problem for communication skills training in the future, due to the increasing number of students, and the time and financial costs related to faculty training and renewal, and the use of simulated patients. The fact that basic sciences-oriented universities may also start to provide medical training soon was seen as a major threat to the maintenance and further development of integrated and longitudinal communication training (Quote 8).

Some participants believed that teaching was still considered less valuable than scientific research when it comes to career and academic promotion, and mentioned that it might be problematic to motivate, train and retain clinicians for communication training activities (Quote 9).

Finally, some participants added that focusing too much on OSCEs to assess communication skills could lead to a reductionist view of communication, with too much emphasis on techniques and checklists, at the expense of authenticity, and other dimensions of the patient-physician relationship (Quote 10).

## Discussion

Our study shows that in 2015, all five Swiss medical schools had a structured and partly continuous communication curriculum. The topics covered during the medical training matched up to two-thirds of the SCLO communication-related learning objectives. Experiential-based instructional and assessment methods were mainly used, but in different proportions, and teachers from various backgrounds taught in all areas of the curricula. However, there was no clear correlation between candidates’ degree of preparedness for communication in professional life and the communication curriculum structure of their medical school. The gap remained in the continuity between pre-clinical, clinical and postgraduate communication training. Also, maintaining the quality, uniformity and coherence of communication teaching continued to be a major challenge. The existence of the FLE, which explicitly includes the assessment of communication skills, was perceived as an opportunity to further improve the communication curricula, and to develop a common framework for teaching and assessing communication, but there was a risk that it could reduce communication training to a list of techniques and other points to be checked off.

The fact that the SCLO was used to set up the communication skills to be assessed in the FLE positively influenced the recognition and formalization of communication as a set of trainable and assessable skills in some medical schools. Given the fact that an undergraduate medical curriculum in Europe includes a total of 5500 h [31], 60 to 200 h of structured curriculum represents only 1.1 to 3.6% of the whole medical program. These results are in line with a British survey showing that the percentage of curriculum time devoted to communication skills training each year ranged from 0.15 to 5.5% [24]. Given that communication skills are often acquired informally during the clinical years, it is not surprising that several communication related-SCLO topics were reported as not covered, or insufficiently covered in most medical schools in our survey. Despite its increasing relevance, emphasis on interprofessional communication in undergraduate medical education is somewhat recent [32]. Shared decision making remains a complex issue which requires both relationship skills, and risk communication skills. Although some programs have been developed in undergraduate training [33, 34], this topic largely remains only a part of the continuous medical education curricula [35].

Several elements of the communication curricula organization of the five medical schools matched international recommendations, such as integration of clinical skills with other skills, and the use of experiential instruction methods [17, 36]. However, although all the Swiss medical schools have a longitudinal communication curriculum, most of the structured training activities took place in the first 3 years, and were often related to pre-clinical study content. In some of the medical schools, the frequency of OSCEs was low compared to that of other countries, like the United Kingdom [37], and the number of stations required to meet the psychometric requirements was not reached [38]. Workplace-based assessment was reported by the teachers and coordinators, but little was known about the importance given to communication issues and the quality of feedback provided in such assessments. A lack of, or insufficient training assessment during the clinical training has recently been pointed out in German-speaking countries [25]. Although the clinical environment is considered to be the best place to teach communication skills in an integrated way [21, 39, 40], the teaching of relationship and communication skills in clinical practice remains highly variable and implicit, and students often report that role modelling in the clinical environment does not reflect the kind of communication taught during the pre-clinical and formal training [41, 42].

The fact that most Swiss medical schools involved teachers from different backgrounds and medical fields in their communication curricula is encouraging, but teachers come from fields where communication is already considered important (general practice, psychosomatics, psychiatry and education). It is important to extend communication skills training and include teachers from a larger number of medical fields, as this conveys the message that good clinical communication is important for patient care in any medical discipline [43]. However, the difficulty of ensuring both quality and homogeneity in communication teaching in clinical practice was reported by several teachers. Teaching communication skills is complex, and the way this subject should be taught and assessed is still debated [44]. However, sharing a common vision of what and how communication should be taught and assessed may lead to better quality teaching. This can be best achieved through faculty development programs [45–47], which focus on reaching a consensus about what content should be taught, developing the ability to recognize good or poor skill performance, and giving constructive and interactive feedback [48].

The introduction of more complex communication topics in the FLE may respond to the challenges and difficulties highlighted in this survey. Firstly, it may encourage curriculum coordinators to increase the number of curricular hours devoted to communication skills, and secondly, it may help bridge the gap between the pre-clinical and clinical years, since complex communication skills are best addressed during the clinical years of training. It may also facilitate the adoption of common frames of reference for all five medical schools, since the assessment of complex communication skills will require the use of more specific assessment scales. In turn, teachers will need to adapt their teaching to these frames of reference, to fulfil the FLE requirements.

This was a national study, involving different key stakeholders from each medical school and using a mixed method approach, providing both quantitative and qualitative data. However, it has several limitations. Less than half of the medical candidates participated, and only key communication teachers were involved in the survey, limiting the generalizability of our results. However, the fact that the graduates’ perceptions on what was taught matched those of the teachers is reassuring. The analysis of communication curricula was based on the written material sent by the curriculum coordinators and communication teachers. The definition of what constitutes communication and communication training activities was left for each medical school to decide, and it is possible that the differences in the number of hours dedicated to structured communication training activities among the five medical schools may be partly explained by differences in perception and definitions of communication training. It was not possible to differentiate between structured and less structured forms of training, especially during clerkships. Furthermore, the analysis of the semi-structured interviews was conducted in a rather straightforward way and we did not expect to reach a point of saturation or articulate our findings to a theoretical framework or model. Finally, the questionnaire used was self-developed and was only partially validated – validity evidence was only sought for the content and response process.

## Conclusion

This first step in a project aiming at improving the assessment of communication skills at the FLE allowed us to see the extent to which the communication curricula at the five Swiss medical schools covered the SCLO and European consensus statements related to communication issues, and matched the recommendations regarding training and assessment. It also helped give a better understanding of the challenges medical schools face regarding communication training and assessment. More efforts should be made to promote training and assessing of complex skills during the clinical years of training, to bridge the gap between the pre-clinical and clinical years and match the SCLO communication issues.

The next steps of this national project – organising a national symposium with international communication experts, and developing more specific communication stations to be used in future FLE sessions - may well represent the best way to facilitate the adoption of common frameworks of reference, and harmonize visions of communication training/teaching and assessment in Swiss medical schools.

## Additional file

Additional file 1: Medical graduates’ perceptions regarding communication skills addressed during the undergraduate curriculum per medical school and preparedness for communication in future professional life. (DOCX 19 kb)

## Abbreviations

EPA: Entrustable professional activities
FLE: Federal licensing examination
MCQ: Multiple choice questions
Mini CEX: Mini clinical evaluation exercise
OSCE: Objective structured clinical encounter
SCLO: Swiss catalogue of learning objectives
SD: Standard deviation
SWOT: Strengths, weaknesses, opportunities, threats
