# Supplementary material for: How do Swiss medical schools prepare their students to become good communicators in their future professional careers: a questionnaire and interview study involving medical graduates, teachers and curriculum coordinators
Source: BMC Med Educ. 2018 Nov 29;18:285. doi: 10.1186/s12909-018-1376-y (PMC6267086; doi:10.1186/s12909-018-1376-y)
Supplement: Supplementary file 1 — Medical graduates’ perceptions regarding communication skills addressed during the undergraduate curriculum per medical school and preparedness for communication in future professional life. (DOCX 19 kb) [file 12909_2018_1376_MOESM1_ESM.docx]

Additional file 1

Medical graduates’ perceptions regarding communication skills addressed during the undergraduate curriculum per medical school and preparedness for communication in future professional life

| Topics of the Swiss catalogue of learning objectives | Graduates’ perceptions. % Yes (responding)* | | | | |  |
| --- | --- | --- | --- | --- | --- | --- |
| **Generally covered topics** | 1 | 2 | 3 | 4 | 5 |  |
| Patient-centered history taking |  |  |  |  |  |  |
| -       Focused | 90.2 (102) | 97.5 (79) | 95.3 (107) | 93.2 (44) | 94.5 (55) |  |
| -       Reasons | 97.0 (99) | 98.7 (77) | 98.1 (106) | 100.0 (43) | 96.5 (57) |  |
| -       Expectations | 70.1 (87) | 94.3 (70) | 93.2 (103) | 97.6 (41) | 87.0 (54) |  |
| -       Somatic-psychological aspects | 89.5 (95) | 97.3 (73) | 98.1 (105) | 97.4 (39) | 100.0 (51) |  |
| -       Concerns | 68.1 (94) | 97.3 (75) | 94.1 (102) | 100.0 (41) | 92.5 (53) |  |
| -       Socio-cultural background | 65.2 (89) | 91.8 (73) | 84.5 (97) | 97.6 (41) | 94.4 (54) |  |
| -       Illness experience | 50.0 (80) | 88.4 (69) | 83.0 (94) | 95.1 (41) | 86.8 (53) |  |
| Ask patient needs regarding health problems | 45.3 (75) | 82.9 (70) | 82.8 (93) | 82.4 (34) | 77.1 (48) |  |
| Disease-illness concepts | 72.3 (83) | 93.9 (66) | 87.0 (100) | 95.0 (40) | 90.2 (51) |  |
| React to nonverbal cues | 48.1 (77) | 86.6 (67) | 84.8 (92) | 91.7 (36) | 78.3 (46) |  |
| Balance proximity-distance | 30.8 (78) | 62.3 (61) | 78.6 (84) | 80.6 (36) | 73.3 (45) |  |
| Show empathy | 65.4 (81) | 98.5 (66) | 90.1 (91) | 97.2 (36) | 93.2 (44) |  |
| Establish and maintain an empathetic relationship | 38.5 (78) | 91.3 (69) | 81.2 (85) | 94.1 (34) | 85.1 (47) |  |
| Give time to patient | 97.9 (97) | 98.7 (76) | 98.1 (104) | 100.0 (37) | 96.4 (55) |  |
| Active listening | 88.3 (94) | 100.0 (75) | 99.0 (102) | 100.0 (40) | 96.2 (53) |  |
| Collecting information about delicate issues (sexual) | 61.8 (89) | 76.4 (72) | 98.1 (103) | 80.0 (40) | 73.1 (52) |  |
| Obtaining patient consent | 58.0 (81) | 86.1 (72) | 90.6 (96) | 91.4 (35) | 94.2 (52) |  |
| Explaining reasons for investigation, risks, advantages,… | 67.5 (80) | 91.4 (70) | 91.6 (95) | 86.1 (36) | 76.5 (51) |  |
| Explaining and checking patient understanding | 57.0 (79) | 95.7 (70) | 91.4 (93) | 94.3 (35) | 95.7 (47) |  |
| Breaking bad news | 45.5 (77) | 98.6 (73) | 92.7 (96) | 79.4 (34) | 87.8 (49) |  |
| Counselling skills | 92.7 (82) | 79.4 (68) | 88.0 (92) | 91.7 (36) | 91.7 (48) |  |
| **Generally less covered topics** |  |  |  |  |  |  |
| History taking in absence of patient | 51.0 (98) | 75.3 (77) | 79.6 (103) | 50.0 (40) | 40.4 (52) |  |
| History taking with special needs patients (dying) | 25.8 (89) | 70.0 (70) | 85.9 (99) | 54.5 (33) | 56.6 (53) |  |
| Communicating with patients with language problems | 12.2 (90) | 23.4 (64) | 26.0 (100) | 23.5 (34) | 24.1 (54) |  |
| Communicating with allophone patients | 19.8 (91) | 30.0 (70) | 57.0 (100) | 82.1 (39) | 64.7 (51) |  |
| Communicating with vulnerable patients | 58.0 (88) | 66.2 (65) | 62.9 (97) | 68.4 (38) | 72.5 (51) |  |
| Phone conversation | 67.0 (94) | 23.6 (72) | 97.1 (104) | 7.3 (41) | 20.0 (55) |  |
| Document information | 25.3 (83) | 40.9 (66) | 38.9 (90) | 65.7 (35) | 45.1 (51) |  |
| Shared decision making | 38.0 (79) | 70.0 (70) | 78.0 (91) | 76.5 (34) | 79.2 (48) |  |
| Use of visual support | 33.8 (77) | 68.2 (66) | 48.3 (87) | 62.1 (29) | 40.8 (49) |  |
| Managing unsatisfied or unhappy patients/families | 13.3 (83) | 37.9 (66) | 75.0 (92) | 54.8 (31) | 33.3 (48) |  |
| Interprofessional communication | 27.6 (76) | 45.6 (68) | 71.4 (91) | 51.4 (35) | 40.4 (47) |  |
| Presentation skills | 60.3 (73) | 61.5 (65) | 68.1 (91) | 66.7 (36) | 51.0 (49) |  |
| **Preparedness for communication in future professional life** | Graduates’ perceptions (Likert 1-5, 1=low; 5=high) | | | | |  |
|  | Mean (SD) | Mean (SD) | Mean (SD) | Mean (SD) | Mean (SD) | p |
|  | 3.60 (0.83) | 4.05 (0.6) | 3.90 (0.75) | 3.96 (0.93) | 3.79 (1.06) | 0.007 |

*Candidates responding ranged from 27 to 107 per medical school (numbers in brackets). Medical schools numbered from 1 – 5
